# Meta-analysis of the safety of voriconazole in definitive, empirical, and prophylactic therapies for invasive fungal infections

**DOI:** 10.1186/s12879-017-2913-8

**Published:** 2017-12-28

**Authors:** Yuanming Xing, Lu Chen, Yan Feng, Yan Zhou, Yajing Zhai, Jun Lu

**Affiliations:** 1 Clinical Research Center, The First Affiliated Hospital of Xi’an Jiaotong University, Xi’an, 710061 China; 2 Hou Zonglian medical experimental class of 2014, Xi’an Jiaotong University, Xi’an, 710061 China; 3 Department of Pharmacy, The First Affiliated Hospital of Xi’an Jiaotong University, Xi’an, 710061 China; 4 Hou Zonglian medical experimental class of 2015, Xi’an Jiaotong University, Xi’an, 710061 China

**Keywords:** Voriconazole, Invasive fungal infections, Tolerability, Meta-analysis

## Abstract

**Background:**

Voriconazole has been used in the treatment and prophylaxis of invasive fungal infections (IFIs) while its wide use was limited by some frequent adverse events, especially neurotoxicity, hepatotoxicity and even renal disruption. The aim of this study was to comprehensively compare voriconazole-induced toxicity, including tolerability, neurotoxicity, visual toxicity, hepatotoxicity and nephrotoxicity with the composite of other antifungals commonly used in clinic.

**Methods:**

Bibliography databases were searched to select randomized controlled trials providing information about the incidence of toxicity referred above. A total of 4122 patients from 16 studies were included in the meta-analysis.

**Results:**

Analysis of individual types of toxicity showed that there was a significant difference between voriconazole and the composite of other antifungal agents. The primary outcome, the tolerability of voriconazole was slightly inferior (OR = 1.71, 95% CI = 1.21–2.40, *P* = 0.002) and it is noteworthy that the probabilities of neurotoxicity and visual toxicity were around twice higher and six-fold for voriconazole compared with the counterpart (OR = 1.99, 95% CI = 1.05–3.75, *P =* 0.03 and OR = 6.50, 95% CI = 2.93–14.41, *P <* 0.00001, respectively). Hepatotoxicity was more common in voriconazole group (OR = 1.60, 95% CI = 1.17–2.19, *P* = 0.003) whereas its pooled risk of nephrotoxicity was about half of the composite of other five antifungal agents (OR = 0.46, 95% CI = 0.26–0.84, *P* = 0.01).

**Conclusion:**

Our analysis has revealed differences in multiple types of toxicity induced by VRC versus other antifungals and quantified the corresponding pooled risks, which could provide an alternative for patients with a certain antifungal intolerance and help the clinician to select the optimal intervention.

**Electronic supplementary material:**

The online version of this article (10.1186/s12879-017-2913-8) contains supplementary material, which is available to authorized users.

## Background

Invasive fungal infections (IFIs) remain a leading cause of morbidity and mortality in immunodeficient critically ill patients. Prophylaxis usually recommended for those patients with unidentified diagnosis but with high risk factors. Empirical antifungal therapy is usually suggested to treat probable and possible IFIs. A definitive therapy is permitted only for the definitive diagnosis as proven fungal infection. The abovementioned three levels of IFI diagnosis were defined according to the EORTC/MSG criteria [1].

Amphotericin B (AMB), triazoles including itraconazole, voriconazole (VRC), posaconazole, isavuconazole as well as combination antifungal therapy with voriconazole and an echinocandin are preferred agents for treatment and prevention of invasive aspergillosis in most patients [2]. Fluconazole and echinocandin including caspofungin, micafungin and anidulafungin are usually recommended for invasive candidiasis while some triazole and AMB are also recommended if there is intolerance limited availability, or resistance to other antifungal agents [3].

Voriconazole is a second-generation triazole antifungal agent that is effective against an IDSA diverse array of fungi, including *Candida*, *Aspergillus*, *Scedosporium*, and *Fusarium* spp. It is recommended as the first-line treatment for invasive aspergillosis by the Infectious Diseases Society of America [2, 4] and is also used in definitive and prophylatic therapies for *Candida* spp. and other molds [3, 5]. However, the universal use of this agent has been restricted by the high frequency of adverse events such as neurotoxicity, visual toxicity, and hepatotoxicity and the potential for nephrotoxicity caused by a cyclodextrin-based vehicle in its intravenous formulation; any of these types of toxicity may lead to discontinuation during treatment [6–8].

While individual reviews of different antifungals have been reported [8–15], there has been no systematic comprehensive comparison of the safety of VRC and other antifungal agents. A systematic review from 2010 individually compared the risk of discontinuation and hepatotoxicity of six antifungals in definitive and empirical therapies for IFIs [4]. Due to its indirect parallels between VRC and other antifungal agents as well as the limited toxicity, its findings need to be clarified and updated. We therefore performed a comprehensive meta-analysis of all relevant data in randomized controlled trials (RCTs) in order to gain a better understanding of the main toxicity profiles for VRC and other antifungals used in definitive, empirical, and prophylactic therapies for IFIs.

## Methods

### Search strategy

We conducted a literature search of PubMed, Embase, and the Cochrane Library from their inceptions up to December 15, 2016. An additional manual literature search was performed by checking the reference lists in eligible articles. The MeSH terms used for keyword and text-word searches were “antifungal agents” and “voriconazole”. The search was limited to human studies.

### Selection criteria

**Fig. 1:**
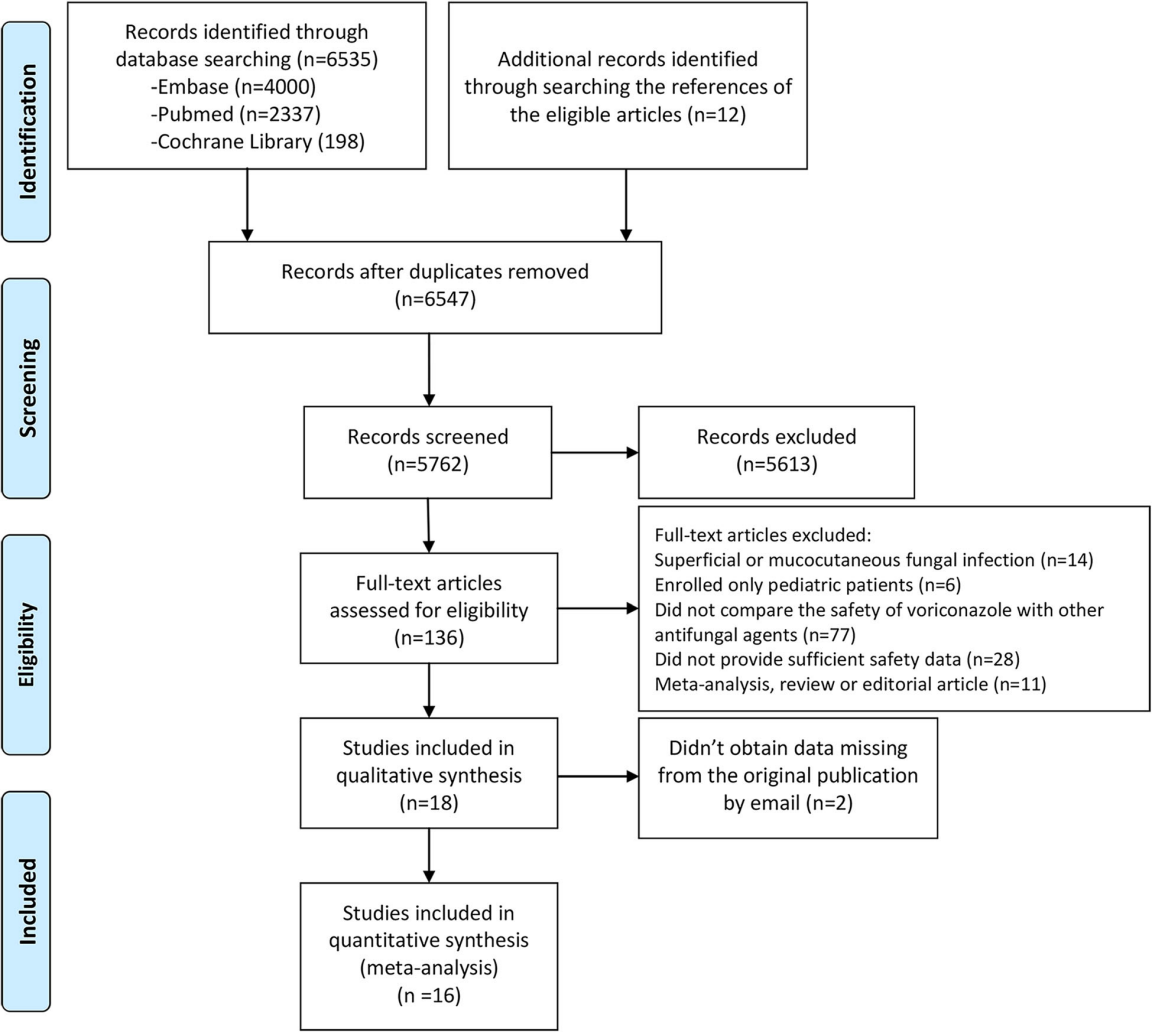
Flow diagram of study selection

Two reviewers independently evaluated each study and identified whether they met the predefined inclusion criteria. We used the PRISMA criteria for searching and selecting studies (Fig. 1). The following inclusion criteria were applied to identify eligible studies: (i) published in the English language before December 15, 2016; (ii) designed as an RCT; (iii) comparing the safety between VRC and other antifungal agents in definitive, empirical, or prophylactic therapy for IFIs; and (iv) providing the incidence of toxicity at least one term among discontinuation, neurotoxicity, visual toxicity, hepatotoxicity, or nephrotoxicity. We excluded studies that enrolled only pediatric patients, studies only focusing on superficial or mucocutaneous fungal infection, and studies of pharmacokinetics, clinical efficacy, and mycology.

### Safety outcomes

The primary outcome was the proportion of patients who discontinued antifungal therapy due to adverse events. The secondary outcome of interest was the cumulative incidence of adverse events related to neurotoxicity, including headache, dizziness, restlessness, hallucination, insomnia and depression and some other nervous system disorders and psychiatric disorders according to version 4.0 of the Common Terminology Criteria for Adverse Events (CTCAE) [16]. Another secondary outcome is visual toxicity which was defined as the occurrence of abnormal vision, photophobia, chromatopsia and some other eye disorders according to CTCAE. We also evaluated the cumulative incidence of laboratory tests of liver dysfunction (serum transaminase, alanine transaminase, alkaline phosphatase, or bilirubin levels) and renal dysfunction (serum creatinine), which represented the safety outcomes of hepatotoxicity and nephrotoxicity. We directly used the laboratory test cutoff values reported for individual studies, since the criteria varied among the different studies.

### Data extraction and quality assessment

Two reviewers independently extracted specific information from studies, including year of publication, authors, study design, therapeutic purposes, antifungals administered, sample size, age, underlying diseases, dose and duration of administration, and safety outcomes including tolerability, neurotoxicity, visual toxicity, hepatotoxicity, and nephrotoxicity. Disagreements about the specific data between two reviewers were resolved through consensus. We attempted to contact the authors by email to seek required data that were missing from the original reports.

Two investigators independently appraised the sources of bias in RCTs according to the Cochrane Collaboration guidelines, and classified each RCT into low, unclear, or high risk by evaluating the following domains: random sequence generation, allocation concealment, blinding of participants and outcome assessment, incomplete outcome data, selective outcome reporting, and other issues [17]. Discrepancies were resolved by discussion with a third investigator. RCTs for which two or more domains were judged as having a high or unclear risk were regarded as having a high overall risk of bias.

### Statistical analysis

We compared the following five safety outcomes between VRC and other antifungal agents: (1) the proportion of antifungal discontinuations due to adverse events, and the cumulative incidences of neurotoxic events related to (2) nervous system and psychiatric disorders and (3) eye disorders (4) liver and (5) renal dysfunction. We performed subgroup analysis to determine if the final results were influenced by certain factors, including different antifungals, therapeutic purpose (definitive, empirical, or prophylactic therapy), and duration (<14 days and ≥14 days). Odds ratios (ORs) and 95% confidence intervals (CIs) were estimated for subgroups with more than two studies. We also performed a sensitivity analysis to examine the stability of the results by excluding a single or multiple studies that were classified by a specific criterion, such as a small sample (*n* < 50), single-center design, or high risk of bias.

A two-sided *P* value of <0.05 was considered indicative of statistical significance. All statistical analyses were performed using RevMan (version 5.3) and Stata (version 12.0, StataCorp, College Station, TX).

## Results

### Study selection and characteristics of included researches

The electronic and manual searches identified 6547 studies, of which 16 RCTs [18–33] involving 4122 randomized patients met the criteria and so were entered into the final analysis (Table 1). A flow diagram of the study selection process is shown in Fig. 1 (see more detailed searching strategy in Additional file 1). The safety outcomes for tolerability, neurotoxicity, visual toxicity, hepatotoxicity, and nephrotoxicity were reported for 12, 10, 13, 14, and 9 studies, respectively. The main underlying disease of patients in the included RCTs was hematological malignancy, followed by solid cancers and other diseases with a high risk of fungal infection. The antifungal agents used in the counterpart groups included fluconazole (*n* = 3), amphotericin B (*n* = 3), itraconazole (*n* = 4), micafungin (*n* = 4), isavuconazole (*n* = 1), and a combination of fluconazole and AMB (*n* = 1). There were eight, three, and five studies that had definitive, empirical, and prophylactic treatment purposes, respectively. The duration of drug therapy was at least 2 weeks in 12 RCTs, less than 2 weeks in 3 RCTs, and unknown in the remaining RCTs.

**Table 1:** Characteristics of included studies

Year	Author	Multicenter	Therapeutic purpose	Antifungals	Sample size	Age	Underlying Disease	Dosing regimen	Duration
2001	Ally	Y	D	VRC	200	18–75	Esophageal Candidiasis, AIDS	200 mg, oral, bid	14d
				FLU	191	18–76	Esophageal Candidiasis, AIDS	200 mg, oral, qd	15d
2002	Lazarus	Y	P	VRC	18	23–61	HM & solid tumor	200/300 mg, oral, q12h	14d
				FLU	6	23–62	HM & solid tumor	400 mg, oral, qd	14d
2002	Herbrecht	Y	D	VRC	144	48.5	HM, SOT, AIDS	200 mg, oral/i.v., bid	77d
				AMBD	133	50.5	HM, SOT, AIDS	1–1.5 mg/kg, i.v., qd	10d
2002	Walsh	Y	E	VRC	415	46.3	FN, HM or other cancers	200 mg, oral, q12h	7d
				LAMB	422	45	FN, HM or other cancers	3 mg/kg, i.v. qd	7d
2005	Kullberg	Y	D	VRC	248	53.6	Candidaemia	200 mg, bid	15d
				AMB/FLU	122	53.3	Candidaemia	AMB 0.7–1.0 mg/kg, oral/i.v., qd,	15d
								Flu, 400 mg, oral/i.v. qd	
2007	Queiroz-Telles	Y	D	VRC	35	48.3	Paracoccidioidomycosis	200 mg, oral, bid	169d
				ITRA	18	48.7	Paracoccidioidomycosis	100 mg, oral, bid	200d
2010	Wingard	Y	P	VRC	305	43	AHSCT	200 mg, oral, bid	100d
				FLU	295	43	AHSCT	400 mg, oral, qd	100d
2010	Kohno.	Y	D	VRC	54	69.9	Tuberculosis sequelae	4 mg/kg, i.v., bid	3w
				MCF	53	72.1	Tuberculosis sequelae	150–300 mg, i.v., qd	3w
2010	Oyake 1	Y	E	VRC	46	NA	FN patients with AML	4 mg/kg, i.v., bid	9d
				MCF	49	NA	FN patients with AML	150 mg, i.v., qd	10d
2011	Bansal	N	D	VRC	15	36.3	CISA	200 mg (adults), 100 mg (children), oral, q12h;	12w
				AMB	18	36.3	CISA	1 mg/kg, i.v., qd	14w
2011	Mattiuzzi	N	P	VRC	71	36.3	AML + MDS	300 mg, i.v., bid	20d
				ITRA	52	60	AML + MDS	200 mg, i.v., qd	21d
2012	Shang	Y	D	VRC	34	37.5	Kidney transplant	4 mg/kg, i.v., q12h	2.9 m
				MCF	31	39.2	Kidney transplant	100 mg (<60 kg), qd;	3.8 m
								150 mg (>60 kg), i.v., qd	
2013	Gao	Y	P	VRC	224	42.3	AHSCT	200/100 mg, oral/i.v., bid	96d
				ITRA	241	42.3	AHSCT	200 mg, oral/i.v., bid	68d
2014	Hayashi	Y	P	VRC	33	NA	AHSCT	200 mg, oral, bid	NA
				ITRA	33	NA	AHSCT	2.5 mg/kg, oral/i.v., bid	NA
2016	Maertens	Y	D	VRC	258	51.2	HM	4 mg/kg/200 mg, oral/i.v., bid	47d
				ISA	258	51.1	HM	200 mg, oral/i.v., qd	45d
2016	Oyake 2	Y	E	VRC	50	53	FN with hematopathy	4 mg/kg, i.v., bid	9d
				MCF	50	53	FN with hematopathy	150 mg, i.v., qd	12d

*Y* Yes, *N* No, *NA* not available, *D* definitive treatment, *E* empirical treatment, *P* prophylaxis, *VRC* voriconazole, *FLU* fluconazole, *AMBD* amphotericin B deoxycholate, *LAMB* liposome amphotericin B, *AMB* amphotericin B, *ITRA* itraconazole, *MCF* micafungin, *ISA* isavuconazole, *HM* hematological malignancy, *SOT* solid organ transplantation, *FN* febrile neutropenia, *AHSCT* allogeneic hematopoietic stem cell transplantation, *AML* acute myeloid leukemia, *CISA* chronic invasive sinus aspergillosis, *MDS* myelodysplastic syndrome

### Bias assessment

**Fig. 2:**
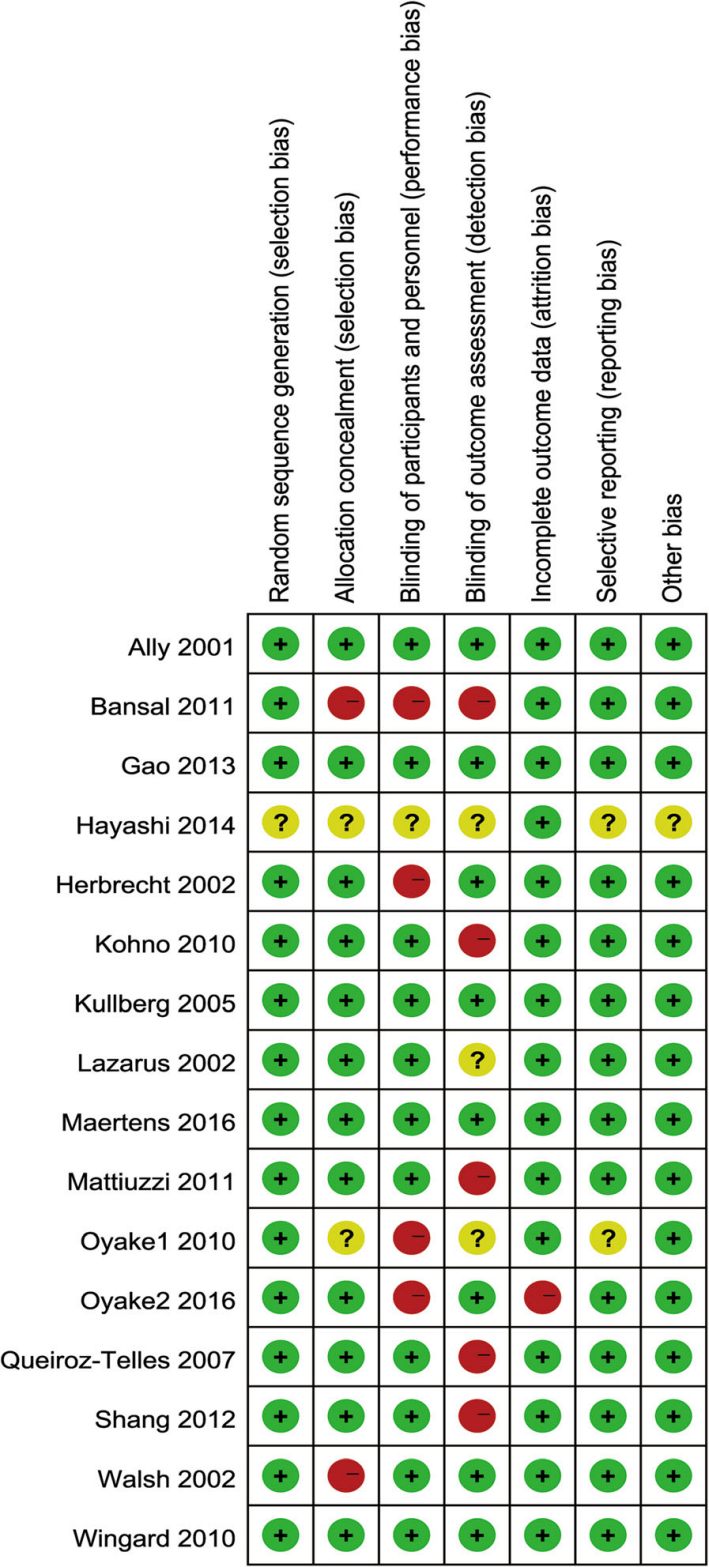
Risk of bias summary: review authors’ judgments about each risk of bias item for each included study

Figure 2 presents the results of the systematic bias analysis. There was a low risk of bias for most items, except for the presence of performance and detection bias due to the lack of a double-blind design and blind outcome assessment in four studies [18, 22, 27, 32].

### Safety outcomes

#### Tolerability

**Fig. 3:**
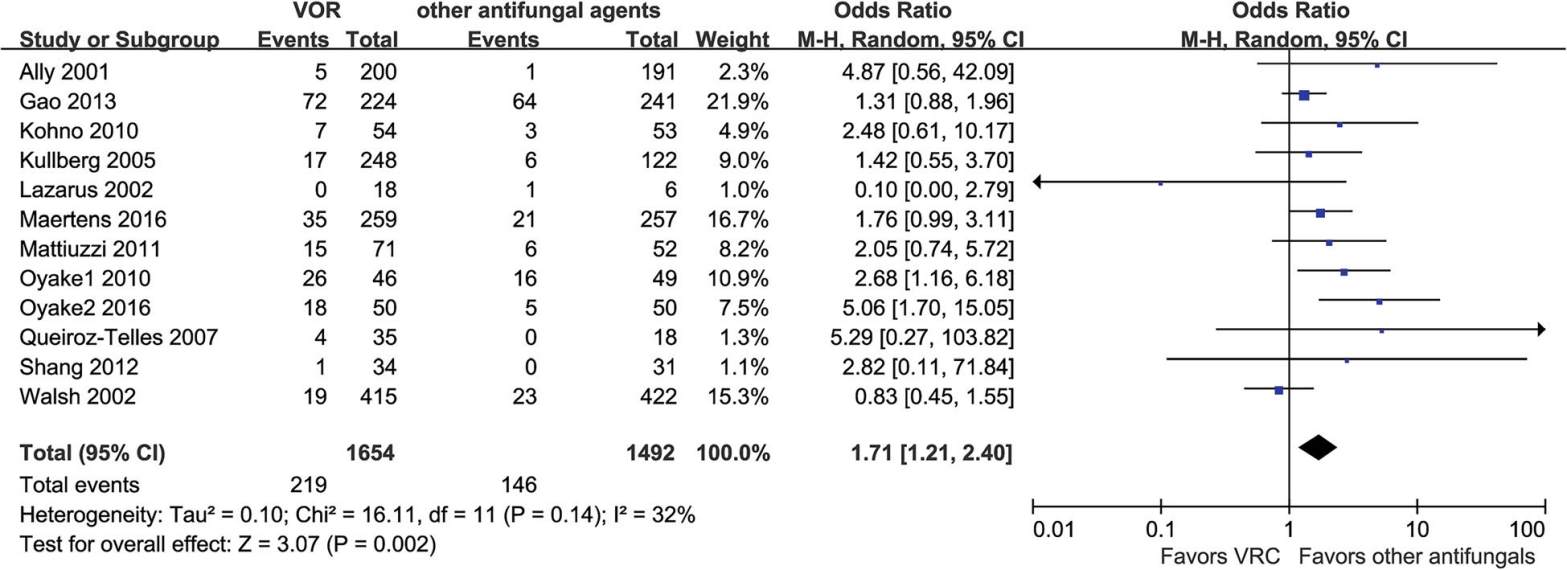
Meta-analysis for tolerability among patients treated with voriconazole and the composite of other antifungal agents

Tolerability could be analyzed for 12 RCTs involving 2146 patients [20, 21, 23, 24, 26–33]. VRC was associated with a higher probability of discontinuation due to adverse events compared with the composite of other antifungal agents (13.2% vs. 9.8%) (OR = 1.71, 95% CI = 1.21–2.40, *P* = 0.002; *I* ^2^ = 32%) (Fig. 3).

#### Neurotoxicity

**Fig. 4:**
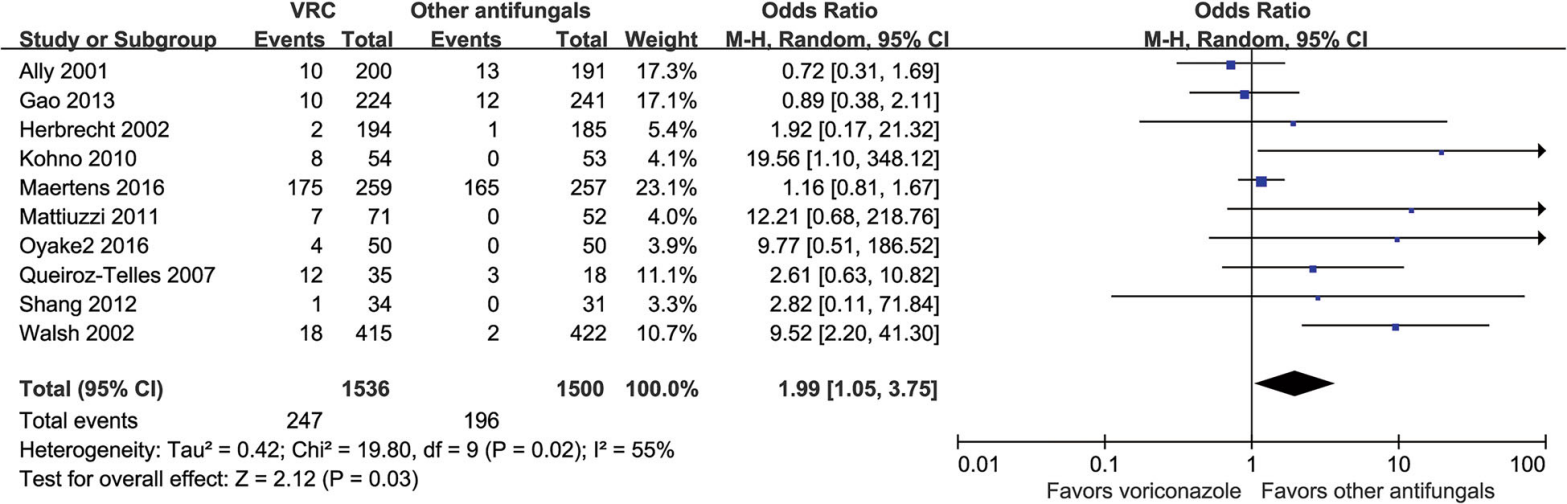
Meta-analysis for neurotoxicity among patients treated with voriconazole and the composite of other antifungal agents

Neurotoxicity could be analyzed for 10 RCTs involving 3036 patients [19, 21, 24, 26, 28–33]. Compared with other antifungal agents, the use of VRC significantly increased the probability of neurotoxicity, from 13.1% to 16.1% (OR = 1.99, 95% CI = 1.05–3.75, *P* = 0.03; *I* ^2^ = 55%) (Fig. 4).

#### Visual toxicity

**Fig. 5:**
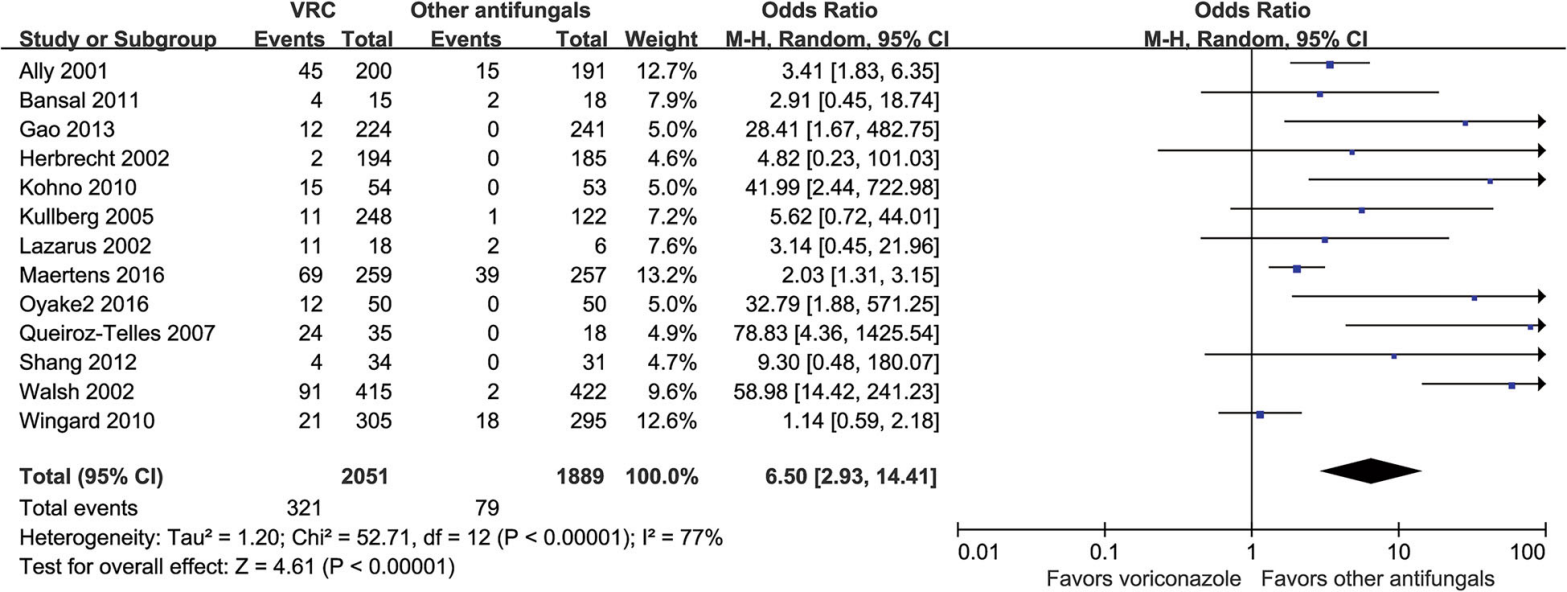
Meta-analysis for visual toxicity among patients treated with voriconazole and the composite of other antifungal agents

Visual toxicity could be analyzed for 13 RCTs involving 3940 patients [19–26, 28, 29, 31–33]. The pooled risk of an incidence of visual toxicity was 15.7% in the VRC group and 4.2% in the counterparts group (comprising combinations of various antifungals) (OR = 6.50, 95% CI =2.93–14.41, *P <* 0.00001; *I* ^2^ = 77%) (Fig. 5).

#### Hepatotoxicity

**Fig. 6:**
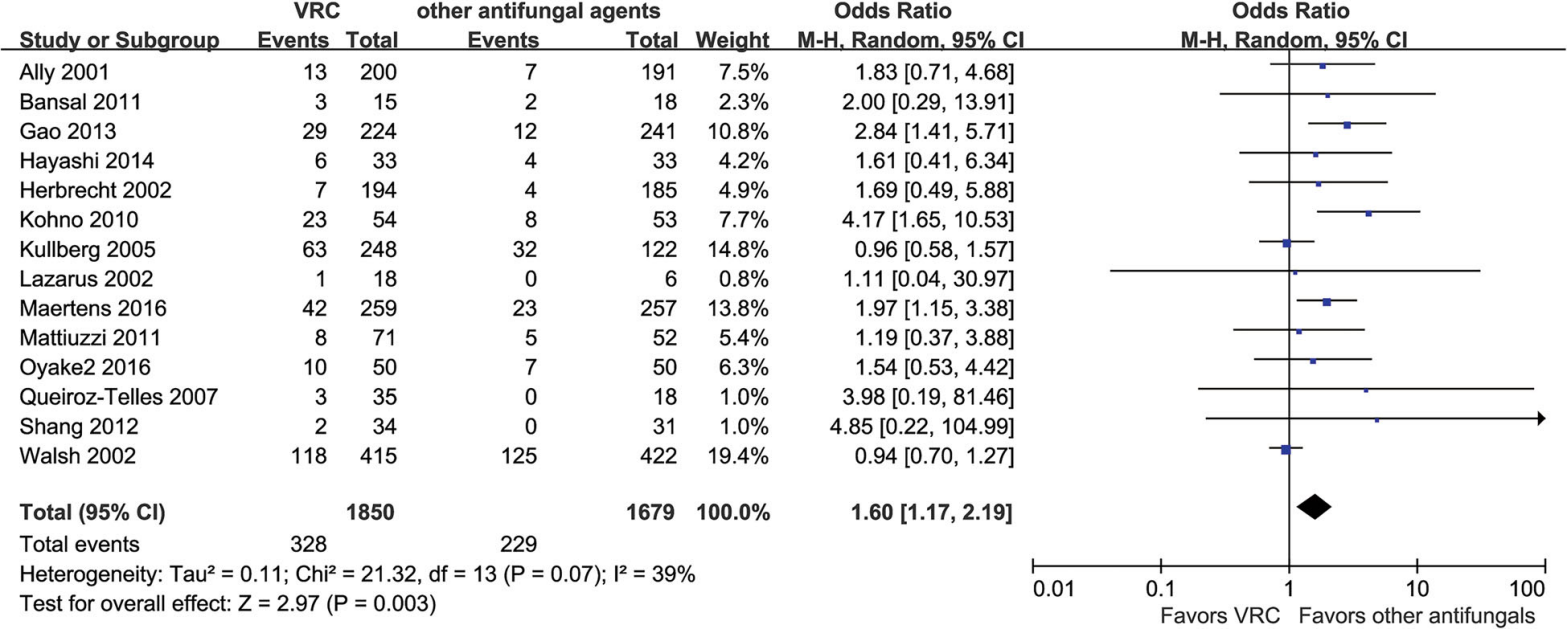
Meta-analysis for hepatotoxicity among patients treated with voriconazole and the composite of other antifungal agents

Hepatotoxicity could be analyzed for 14 RCTs involving 3529 patients [18–24, 26, 28–33]. The pooled risk of an elevation in liver enzyme levels was 13.6% in the VRC group and 17.7% in the counterparts group (comprising combinations of various antifungals) (OR = 1.60, 95% CI =1.17–2.19, *P* = 0.003; *I* ^2^ = 39%) (Fig. 6).

#### Nephrotoxicity

**Fig. 7:**
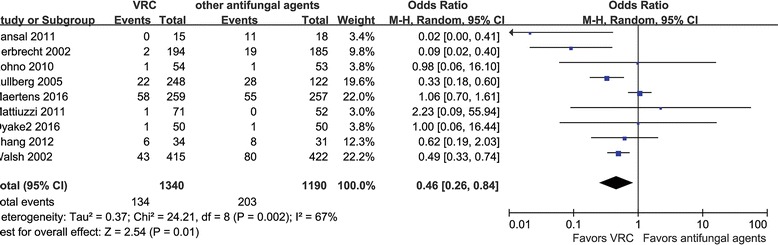
Meta-analysis for nephrotoxicity among patients treated with voriconazole and the composite of other antifungal agents

Nephrotoxicity could be analyzed for 9 RCTs involving 2530 patients [20–22, 28–32]. The rate of the abnormal renal function was significantly lower in the VRC group than in the counterparts group (10.0% vs. 17.1%) (OR = 0.46, 95% CI = 0.26–0.84, *P* = 0.02; *I* ^2^ = 67%) (Fig. 7).

### Subgroup analysis

In a further subgroup analysis by types of antifungal agents, therapeutic purpose, and duration, the risk of adverse events differed between treatment with VRC and other antifungals (Table 2).

**Table 2:** Summary of subgroup analysis for the safety of voriconazole and other antifungals

Outcome	Subgroup	Variables	No. of studies	No. of patients		OR (95% CI)	*I* ^*2*^%	*P*
				VRC	counterpart			
Tolerability	Antifungals	FLU	2	5/218	2/197	0.86 (0.02, 38.38)	73	0.94
		AMB	1	19/415	23/422	0.83 (0.45, 1.55)	NA	0.56
		ITRA	3	91/330	70/311	1.42 (0.98, 2.06)	0	0.06
		MCF	4	52/184	24/183	3.19 [1.77, 5.76]	0	0.0001
		ISA	1	35/259	21/257	1.76 (0.99, 3.11)	NA	0.05
	Therapeutic purpose	definitive	6	69/830	31/672	1.87 [1.20, 2.92]	0	0.005
		empirical	3	63/511	44/521	2.10 (0.72, 6.15)	80	0.18
		prophylaxis	3	87/313	71/299	1.35 (0.67, 2.69)	34	0.40
	Duration	<14d	3	63/511	44/521	2.10 (0.72, 6.15)	80	0.18
		≥14d	9	156/1143	102/971	1.54 [1.16, 2.05]	0	0.003
Neurotoxicity	Antifungals	FLU	1	10/200	13/191	0.72 [0.31, 1.69]	NA	0.45
		AMB	2	20/609	3/607	6.87 [2.04, 23.16]	21	0.002
		ITRA	3	29/330	15/311	1.62 [0.84, 3.14]	52	0.15
		MCF	3	13/138	0/134	10.28 [1.91, 55.44]	0	0.007
		ISA	1	175/259	165/257	1.16 [0.81, 1.67]	NA	0.42
	Therapeutic purpose	definitive	6	208/776	182/735	1.27 [0.93, 1.73]	26	0.13
		empirical	2	22/465	2/472	9.57 [2.57, 35.60]	0	0.0008
		prophylaxis	2	17/295	12/293	1.40 [0.66, 2.97]	69	0.39
	Duration	≥14d	8	225/1071	194/1028	1.29 [0.97, 1.72]	29	0.08
		<14d	2	22/465	2/472	9.57 [2.57, 35.60]	0	0.0008
Visual toxicity	Antifungals	FLU	3	77/523	35/492	2.11 [1.38, 3.24]	66	0.0006
		AMB	3	97/624	4/625	28.83 [10.84, 76.64]	75	<0.00001
		ITRA	2	37/259	0/259	46.57 [5.89, 368.06]	0	0.0003
		MCF	3	31/138	0/134	26.63 [5.15, 137.62]	0	<0.0001
		ISA	1	69/259	39/257	2.03 [1.31, 3.15]	NA	0.002
	Therapeutic purpose	definitive	8	175/1039	57/875	3.33 [2.41, 4.59]	49	<0.00001
		empirical	2	103/465	2/472	53.85 [15.23, 190.37]	0	<0.00001
		prophylaxis	3	44/547	20/542	1.93 [1.11, 3.36]	68	0.02
	Duration	<14d	11	219/1586	77/1417	2.92 [2.21, 3.85]	57	<0.00001
		≥14d	2	103/465	2/472	53.85 [15.23, 190.37]	0	<0.00001
Hepatotoxicity	Antifungals	FLU	2	14/218	7/197	1.76 (0.71, 4.36)	0	0.22
		AMB	3	128/624	131/625	0.99 (0.74, 1.32)	0	0.95
		ITRA	4	46/363	21/344	2.19 (1.27, 3.76)	0	0.005
		MCF	3	35/138	15/134	2.78 (1.38, 5.60)	4	0.004
		ISA	1	42/259	23/257	1.97 (1.15, 3.38)	NA	0.01
	Therapeutic purpose	definitive	8	156/1039	76/875	1.79 (1.18, 2.71)	29	0.006
		empirical	2	128/465	132/472	0.98 (0.73, 1.30)	0	0.88
		prophylaxis	4	44/346	21/332	2.11 (1.22, 3.63)0	0	0.07
	Duration	<14d	2	128/465	132/472	0.98 (0.73, 1.30)	0	0.88
		≥14d	11	194/1352	93/1174	1.82 (1.31, 2.54)	19	0.0003
Nephrotoxicity	Antifungals	FLU	0	NA	NA	NA	NA	NA
		AMB	3	45/624	110/625	0.15 (0.03, 0.84)	78	0.03
		ITRA	1	1/71	0/52	2.23 (0.09, 55.94)	NA	0.62
		MCF	3	8/138	10/134	0.70 (0.25, 1.94)	0	0.49
		ISA	1	58/259	55/257	1.06 (0.70, 1.61)	NA	0.79
	Therapeutic purpose	definitive	6	89/804	122/666	0.36 (0.14, 0.91)	78	0.03
		empirical	2	44/465	81/472	0.50 (0.34, 0.74)	0	0.0006
		prophylaxis	1	1/71	0/52	2.23 (0.09, 55.94)	NA	0.62
	Duration	<14d	2	44/465	81/472	0.50 (0.34, 0.74)	0	0.0006
		≥14d	7	90/875	122/718	0.41 (0.17, 0.98)	74	0.05

*NA* not applicable

The risk of discontinuation did not differ significantly between VRC and each single individual antifungal except for micafungin, which was associated with significantly reduced odds of discontinuation compared with VRC. According to the subgroup analysis of various treatment purposes, we found that VRC showed a significant poor tolerance in the definitive treatment compared with the empirical and prophylaxis treatment. Concerning the duration of treatment, VRC was more likely to be discontinued than other antifungal agents in patients treated for longer than 14 days. VRC was associated with significantly high risk of neurotoxicity compared with AMB and micafungin while other triazoles including fluconazole, itraconazole and isavuconazole failed to provide a significant reduction or increase in the incidence rate of neurotoxicity. Additionally, we also identified statistically significance in the odd of neurotoxicity in VRC versus other antifungal agents in the subgroup of empirical treatment and duration less than 14 days. Of notes, the studies involved in these two groups are the same, including the counterparts of AMB and micafungin, which may lead to this significant diffference owing to the antifungal type. However, there was no statistically significant difference in the group of definitive and prophylactic treatment as well as duration ≥14 days. The high risk of visual toxicity during VRC treatment was consistent across all analyzed subgroups and the heterogeneity was also reduced attribute to the stratification of three subgroups analysis. For the outcome of hepatotoxicity, VRC showed a higher risk of abnormal liver enzyme levels compared with itraconazole, micafungin, and isavuconazole, but not for fluconazole or AMB. Patients who were diagnosed as definitive IFIs had a high risk of the elevation of liver enzyme levels in the VRC group. In addition, a duration of at least 2 weeks seemed to be a risk factor for liver injury when patients were treated with VRC rather than other antifungals. The heterogeneity of hepatotoxicity was also reduced by stratifying the data by all three subgroups. The occurrence of abnormal renal function did not differ significantly between VRC and the other antifungal agents except AMB, which was associated with a higher risk of nephrotoxicity. The probability of abnormal renal function in definitive and empirical fungal therapies was lower for VRC than for the composite of the other five antifungals. However, the risk of nephrotoxicity was significantly higher for patients treated with other antifungals than for VRC for treatment durations shorter than 14 days.

### Sensitivity analysis

The sensitivity analysis verified the robustness of the findings for each outcome. A leave-one-out sensitivity analysis showed that no individual study excessively influenced the pooled effect in our meta-analysis except for the outcome of neurotoxicity (see Additional file 2: Figs. S1–S5), which was affected by removing 5 single study (including studies of Walsh, Queiroz-Telles, Kohno, Mattiuzzi and Oyake 2 [21, 26, 29, 30, 32]). In addition, the results for each outcome remained stable after excluding RCTs with samples smaller than 50, with a single-center design or with a high risk of bias (see in Additional file 3: Table S1).

## Discussion

This meta-analysis found that the pooled risks of the primary and other four secondary safety outcomes differed significantly between VRC and the composite of other antifungal treatments. VRC was associated with higher risks of treatment discontinuation, neurotoxicity, visual toxicity, and hepatotoxicity but a lower risk of nephrotoxicity compared with the other antifungals.

The combined discontinuation rate of VRC was 13.2% in our meta-analysis. Wang found a discontinuation rate of 9.5%, but this slight discrepancy could have been due to that study involving only three RCTs [4]. According to the results of the present meta-analysis of the primary outcome of tolerability, patients treated with VRC were more likely to discontinue therapy compared with the composite of the other five antifungals. However, the significant difference between these two groups could disappear when they were grouped according to specific drugs. The heterogeneity associated with multiple drugs could explain this change, while the therapeutic purpose and duration of therapy might also have influenced the final results. Patients with definitive IFIs and longer treatment durations were more vulnerable to discontinuation of VRC than the counterparts, which could be due to the severe underlying condition and long-term drug accumulation. Sensitivity analysis based on the small sample size (*n* < 50), single center and high risk of bias did not significantly change the results. However, it should be noted that all of the outcomes in our results need to be interpreted with caution since the counterpart of the VRC group was defined as a composite of antifungal agents rather than a single antifungal.

VRC-associated neurotoxicity, especially hallucination, is a common type of toxicity that is considered to significantly limit its application in the clinic [5, 11, 34]. Our findings indicated that the pooled risk of neurotoxicity for patients treated with VRC was almost twice higher than for those treated with other antifungals. Subgroup analysis indicated that VRC had similar incidence of neurotoxicity with trizoles (excluded of posaconazole) while a higher risk of neurotoxicity compared with AMB and micafungin. Zonios [34] exclusively investigated the incidence of hallucinations associated with VRC therapy, and found a rate of 16.6%, which is similar as our result (16.1% for the pooled incidence of neurotoxicity).

VRC-associated visual disturbance is also a common type of toxicity according to clinical observation [21, 24, 28, 35, 36]. Our main findings indicated that the pooled risk of visual events for patients treated with VRC was 6.5 fold higher than for those treated with other antifungals. Subgroup analysis demonstrated that the incidence of visual disturbance still differed significantly between VRC and other antifungals in each single subgroup even though there were some variations in ORs. The pooled risk of visual toxicity showed the highest gap (15.7% in VRC vs. 4.2% in the counterpart) among all of the observed toxicity outcomes. These two adverse events, neurotoxic and visual adverse events, although usually reversible, often lead to premature discontinuation of VRC [28].

The pooled risk of VRC-associated hepatotoxicity in our analysis (17.7%) was similar to that found by Wang (19.7%) [4]. We found that the risk of hepatotoxicity was higher for VRC than for any combination of the other five antifungals. VRC was involved in occurrence of hepatotoxicity in the definitive treatment group but not in the empirical and prophylactic treatment groups; the severe underlying condition and infectious situation may have contributed to this difference. A longer treatment duration also influenced the risk of hepatotoxicity.

The risk of nephrotoxicity was lower in the VRC group, but only AMB showed a significant higher risk of nephrotoxicity in the subgroup analysis, which was consistent with other studies [6, 37]. Unexpectedly, the nephrotoxicity risk was found to differ significantly between these two groups when the treatment duration was shorter than 14 days [21, 32]. These two studies compared VRC with liposomal AMB and micafungin, respectively. The probability of nephrotoxicity was the same in the RCT comparing VRC and micafungin, meaning that the result was due to the RCT comparing VRC with AMB [21]. A high AMB-induced nephrotoxicity could explain this result. The small number of studies and the combination of heterogeneous antifungals in the counterparts group could be the root cause of the unexpected results and the significant heterogeneity in nephrotoxicity outcomes.

Another newly released triazole antifungal agent, posaconazole, showed similar treatment–related adverse events with fluconazole for prophylaxis in severe Graft-versus-Host Disease [38]. Additionally, patient receiving voriconazole was proved to have more treatment-related adverse events than those receiving fluconazole [39]. Thus posaconazole seems to cause less adverse events than voriconazole according to this indirect evidence. There were also some studies which use posaconazole as a substitution of voriconazole due to its intolerance [40, 41]. However, there is no RCT comparing voriconazole and posaconazole in safety area. Further studies are needed in this respect.

### Strengths and weaknesses

The previous meta-analysis regarding the status of voriconazole among all antifungal agents mostly focused on the efficacy and pharmacoeconomics [42–44]. To our knowledge, this is the first study to have comprehensively compared five common adverse events induced by VRC and other antifungals by systematically reviewing RCTs. The results can be used by clinicians to avoid unnecessary adverse effects and select a better antifungal therapeutic scheme for patients. Furthermore, we also quantified the specific outcomes concerning different antifungals, treatment purposes, and durations by subgroup analysis for four safety outcomes.

Several limitations of our study should be considered. First, as is inevitable for any meta-analysis, our study shares the limitations of the original studies. However, we performed the largest pooled comparison of the toxicity profiles of VRC and a composite of other common antifungal agents based on RCTs. Second, we directly used the criteria applied in each study because the thresholds for abnormal liver and renal enzyme levels varied between them. Finally, the combination of various antifungals in the counterparts group may have been responsible for the moderate heterogeneity and a certain degree of publication bias among the studies (see in Additional file 4: Figure S6). The present findings therefore need to be confirmed in future high-quality studies.

## Conclusion

This meta-analysis provides a comprehensive synthesis of the evidence of harm that voriconazole is associated with higher pooled risk of treatment discontinuation, neurotoxicity, visual toxicity, hepatotoxicity but a lower risk of nephrotoxicity compared with other antifungal agents based on the evidence of current RCTs. The results could point to alternative treatments for a patient with a certain antifungal intolerance, thereby helping the clinician select the optimal intervention.

## Additional files

Additional file 1: Detailed searching strategy. It provided more detail about the searching strategy we used in the present study. (PDF 105 kb)

Additional file 2: Sensitive analysis and the funnel plots under the five outcomes. The sensitive analysis part included the influence of individual study involved in the evaluation of tolerability, neurotoxicity, visual toxicity, hepatotoxicity and nephrotoxicity (see in Figure S1-S5). (PDF 433 kb)

Additional file 3: A summary of sensitive analysis for voriconazole safety after excluding a group of studies which was considered as the potential impact factors to the final results was shown in Table S1. (PDF 33 kb)

Additional file 4: The funnel plots for the tolerability, neurotoxicity, visual toxicity, hepatotoxicity, and nephrotoxicity were presented in Figure S6. (PDF 238 kb)

## Abbreviations

AHSCT: allogeneic hematopoietic stem cell transplantation
AMB: amphotericin B
AMB: amphotericin B
AMBD: amphotericin B deoxycholate
AML: acute myeloid leukemia
Cis: confidence intervals
CISA: chronic invasive sinus aspergillosis
CTCAE: Common Terminology Criteria for Adverse Events
D: definitive treatment
E: empirical treatment
EORTC/MSG: European Organization for Research and Treatment of Cancer/Invasive Fungal Infections Cooperative Group and the National Institute of Allergy and Infectious Diseases Mycoses Study Group
FLU: fluconazole
FN: febrile neutropenia
HM: hematological malignancy
IFIs: invasive fungal infections
ISA: isavuconazole
ITRA: itraconazole
LAMB: liposome amphotericin B
MCF: micafungin
MDS: myelodysplastic syndrome
N: No
NA: not available
OR: Odds ratios
P: prophylaxis
RCTs: randomized controlled trials
SOT: solid organ transplantation
VRC: voriconazole
VRC: voriconazole
Y: Yes
