# Supplementary material for: Meta-analysis of the safety of voriconazole in definitive, empirical, and prophylactic therapies for invasive fungal infections
Source: BMC Infect Dis. 2017 Dec 28;17:798. doi: 10.1186/s12879-017-2913-8 (PMC5745890; doi:10.1186/s12879-017-2913-8)
Supplement: Supplementary file 1 — Detailed searching strategy. It provided more detail about the searching strategy we used in the present study. (PDF 105 kb) [file 12879_2017_2913_MOESM1_ESM.pdf]

Detailed searching strategy

Three database including PubMed, Embase, and the Cochrane Library were searched before December 15, 2016. In order not to miss any relevant literature, we decided to search all related studies with the title containing “voriconazole” and under the classification of “antifungal agents”. The search from PubMed, Embase, and the Cochrane Library were all limited to human studies.

a) Search the MeSH term as “antifungal agents” and the text-word, “voriconazole” in PubMed.
b) Search the Emtree term as “antifungal agents” and the text-word, “voriconazole” in Embase.
c) Search the MeSH term as “antifungal agents” and the text-word, “voriconazole” in the Cochrane Library.
d) Studies were counted after duplicate checking process for each database.
e) An additional manual literature search was performed by checking the reference lists in eligible articles.
