# Supplementary material for: Meta-analysis of the safety of voriconazole in definitive, empirical, and prophylactic therapies for invasive fungal infections
Source: BMC Infect Dis. 2017 Dec 28;17:798. doi: 10.1186/s12879-017-2913-8 (PMC5745890; doi:10.1186/s12879-017-2913-8)
Supplement: Supplementary file 2 — Sensitive analysis and the funnel plots under the five outcomes. The sensitive analysis part included the influence of individual study involved in the evaluation of tolerability, neurotoxicity, visual toxicity, hepatotoxicity and nephrotoxicity (see in Figure S1-S5). (PDF 433 kb) [file 12879_2017_2913_MOESM2_ESM.pdf]

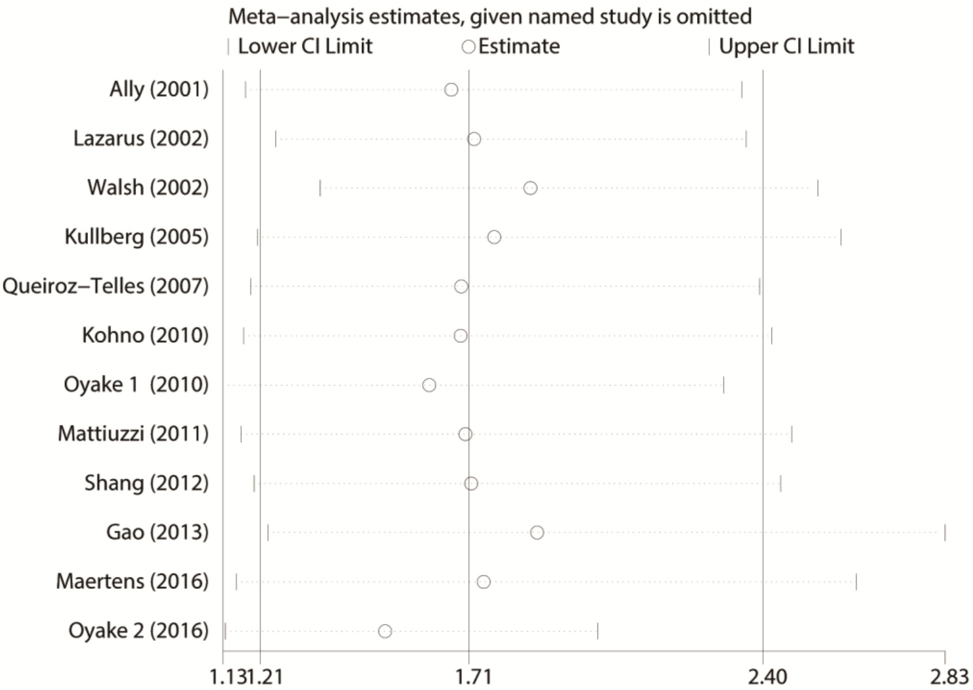


**Figure S1.** Sensitive analysis of individual study involved in the evaluation of tolerability.

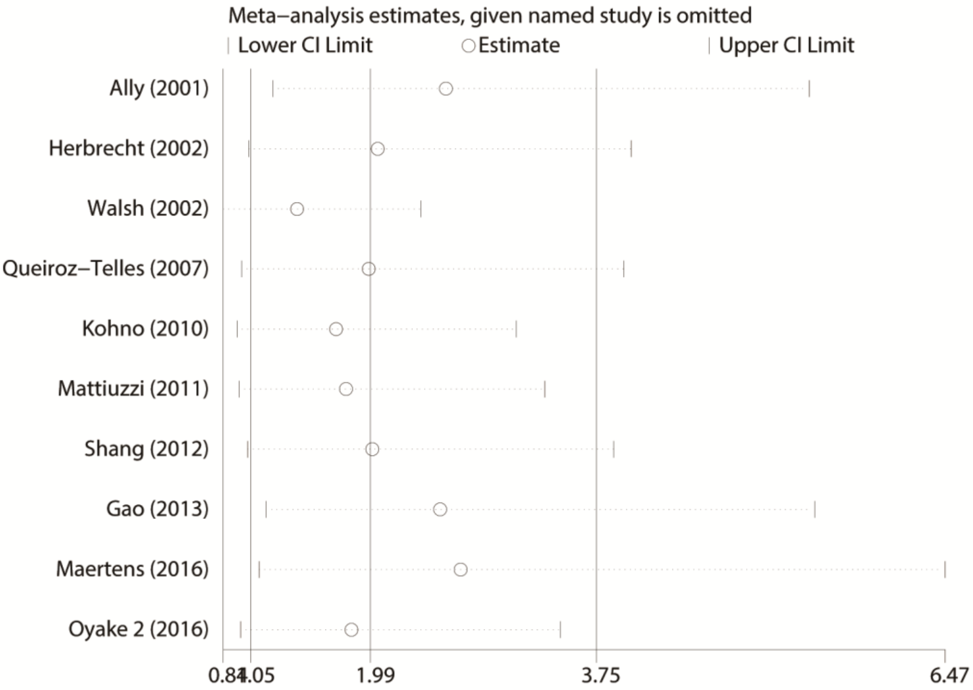


**Figure S2.** Sensitive analysis of individual study involved in the evaluation of

neurotoxicity.

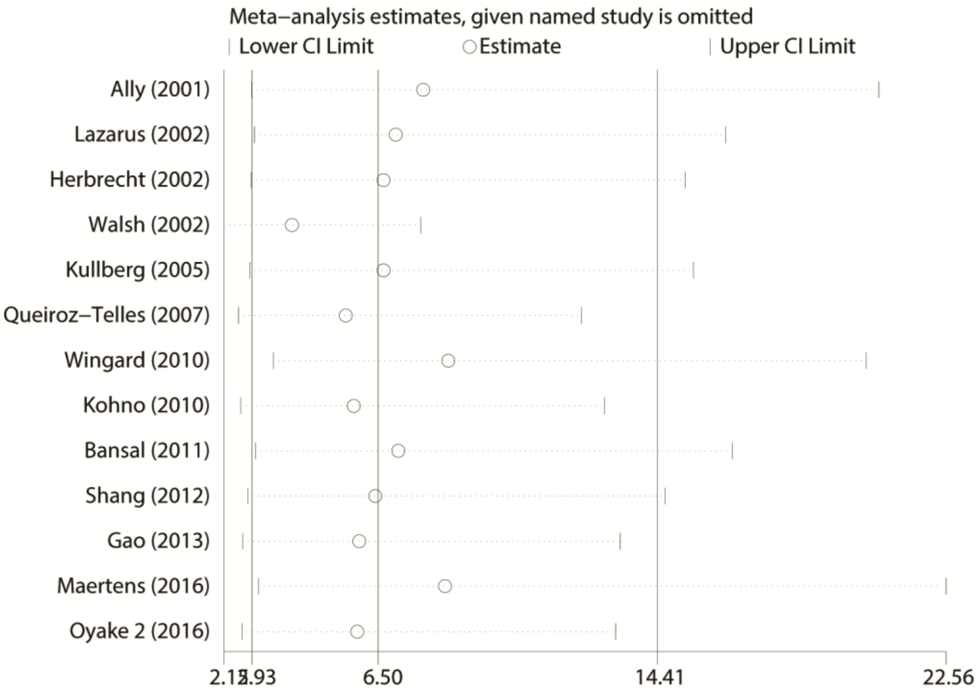


**Figure S3.** Sensitive analysis of individual study involved in the evaluation of visual toxicity.

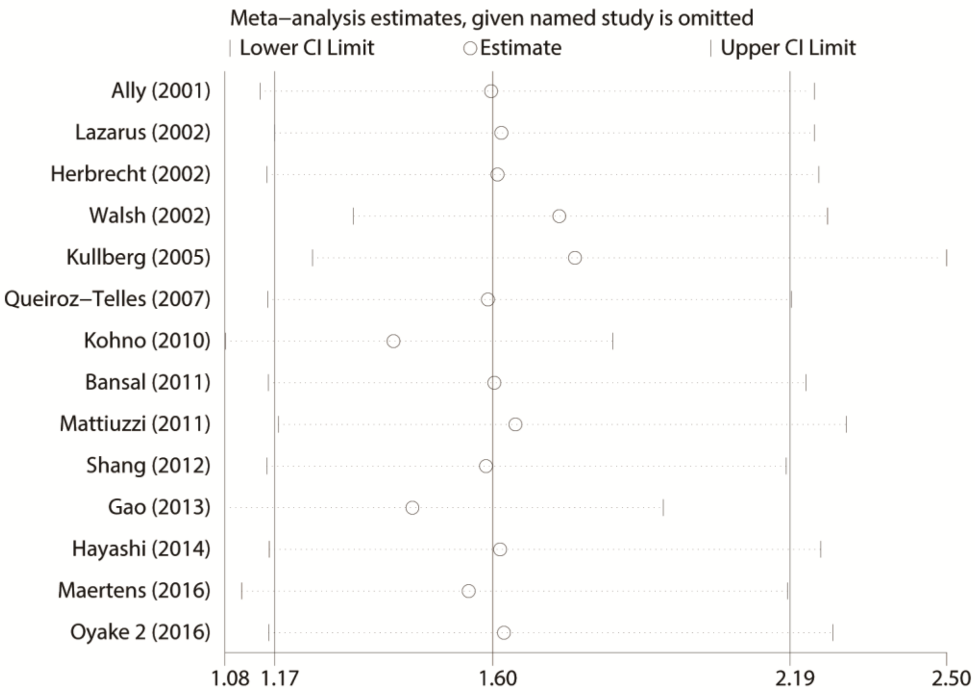

**Figure S4.** Sensitive analysis of individual study involved in the evaluation of hepatotoxicity.

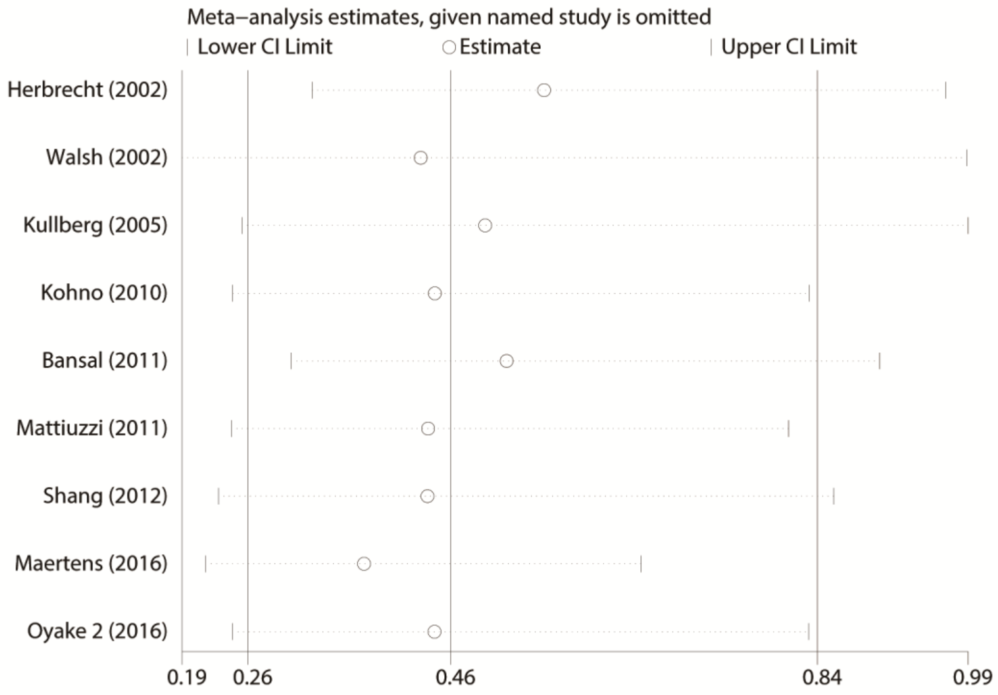


**Figure S5.** Sensitive analysis of individual study involved in the evaluation of nephrotoxicity.
