# Supplementary material for: Meta-analysis of the safety of voriconazole in definitive, empirical, and prophylactic therapies for invasive fungal infections
Source: BMC Infect Dis. 2017 Dec 28;17:798. doi: 10.1186/s12879-017-2913-8 (PMC5745890; doi:10.1186/s12879-017-2913-8)
Supplement: Supplementary file 3 — A summary of sensitive analysis for voriconazole safety after excluding a group of studies which was considered as the potential impact factors to the final results was shown in Table S1. (PDF 33 kb) [file 12879_2017_2913_MOESM3_ESM.pdf]

Table S1. Summary of sensitive analysis for voriconazole safety

Outcomes	Term excluded	Studies excluded	I^2 primary	I^2	OR(95% CI) primary	OR(95% CI)	P primary	P
tolerability	Small sample size (n<50)	Lazarus 2002	32	26	1.71 [1.21, 2.40]	1.72 [1.25, 2.36]	0.002	0.0009
	Single center	Mattiuzzi 2011	32	37	1.71 [1.21, 2.40]	1.70 [1.17, 2.47]	0.002	0.006
	High risk of bias.	Oyake1 2016<br>Oyake2 2016	32	2	1.71 [1.21, 2.40]	1.39 [1.06, 1.82]	0.002	0.02
neurotoxicity	Small sample size (n<50)	NO	55	55	1.52 [1.15, 1.99]	1.52 [1.15, 1.99]	0.03	0.003
	Single center	Bansal 2011	55	53	1.52 [1.15, 1.99]	1.45 [1.10, 1.91]	0.03	0.008
	High risk of bias	Oyake2 2016	55	55	1.52 [1.15, 1.99]	1.47 [1.12, 1.94]	0.03	0.006
visual toxicity	Small sample size (n<50)	Lazarus 2002<br>Bansal 2011	77	81	4.42 [3.42, 5.71]	4.47 [3.44, 5.82]	<0.00001	<0.00001
	Single center	Bansal 2011	77	79	4.42 [3.42, 5.71]	4.45 [3.43, 5.77]	<0.00001	<0.00001
	High risk of bias	Bansal 2011<br>Oyake2 2016	77	80	4.42 [3.42, 5.71]	4.28 [3.29, 5.56]	<0.00001	<0.00001
hepatotoxicity	Small sample size (n<50)	Lazarus 2002<br>Bansal 2011	39	48	1.60 [1.17, 2.19]	1.62 [1.16, 2.27]	0.003	0.005
	Single center	Bansal 2011<br>Mattiuzzi 2011	39	48	1.60 [1.17, 2.19]	1.65 [1.17, 2.34]	0.003	0.005
	High risk of bias	Bansal 2011<br>Hayashi 2014	39	52	1.60 [1.17, 2.19]	1.64 [1.13, 2.38]	0.003	0.009
nephrotoxicity	Small sample size (n<50)	Bansal 2011	67	63	0.46 [0.26, 0.84]	0.52 [0.30, 0.90]	0.01	0.02
	Single center	Bansal 2011<br>Mattiuzzi 2011	67	68	0.46 [0.26, 0.84]	0.50 [0.28, 0.88]	0.01	0.02
	High risk of bias	Bansal 2011<br>Oyake2 2016	67	68	0.46 [0.26, 0.84]	0.51 [0.29, 0.90]	0.01	0.02

NA, not available.
