# Supplementary material for: Meta-analysis of the safety of voriconazole in definitive, empirical, and prophylactic therapies for invasive fungal infections
Source: BMC Infect Dis. 2017 Dec 28;17:798. doi: 10.1186/s12879-017-2913-8 (PMC5745890; doi:10.1186/s12879-017-2913-8)
Supplement: Supplementary file 4 — The funnel plots for the tolerability, neurotoxicity, visual toxicity, hepatotoxicity, and nephrotoxicity were presented in Figure S6. (PDF 238 kb) [file 12879_2017_2913_MOESM4_ESM.pdf]

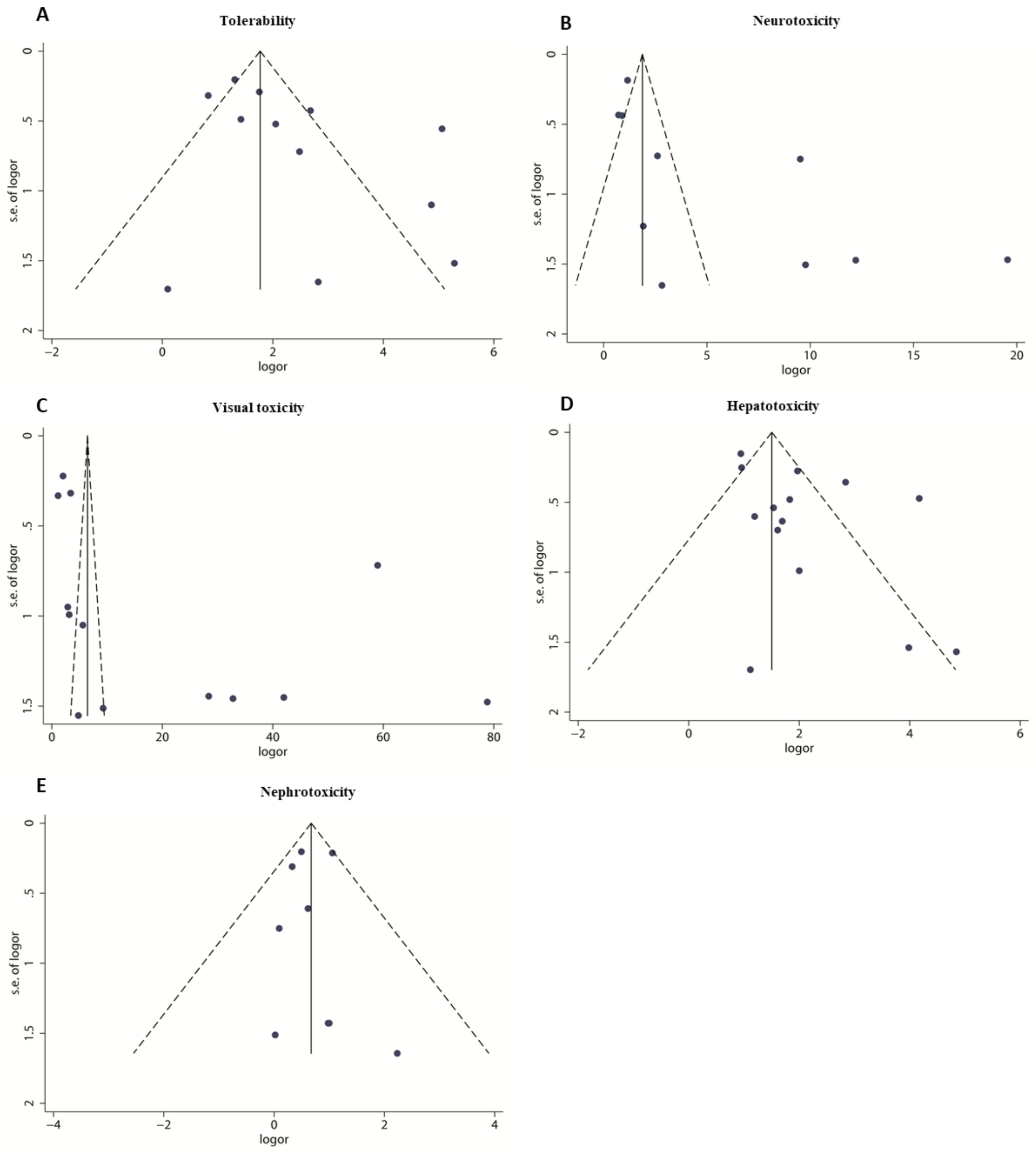

Figure S6. Funnel plots for the tolerability (A), neurotoxicity (B), visual toxicity (C), hepatotoxicity (D), and nephrotoxicity (E).
